# Implementation of automated personalised breast radiotherapy planning techniques with scripting in Raystation

**DOI:** 10.1259/bjr.20220707

**Published:** 2023-02-20

**Authors:** Ian Gleeson, Niall Bolger, Harmony Chun, Katie Hutchinson, Magdalena Klodowska, Jennifer Mehrer, Marian Toomey

**Affiliations:** 1 Department of Medical Physics, Cambridge University Hospitals NHS Foundation Trust, Hills Road, Cambridge, UK

## Abstract

**Objective:**

Implement scripted automatic breast planning (AP) for breast techniques within Raystation.

**Methods:**

Manual plans (MPs) were re-planned and compared with AP plans for whole breast (WB), partial breast (PB), hybrid volumetric modulated arc therapy simultaneous integrated boost (VMAT SIB) and VMAT nodal plans.

**Results:**

WB AP plans took 7 min comparing well to MP. One WB AP failed a mandatory dose constraint. Small statistically significant differences showed improved coverage for AP at expense of slightly hotter plans, however absolute differences were small (mean differences < 1% or D _0.5cc_<0.2 Gy). PB AP plans took 9 min, showing improved coverage (V _24.7Gy_97.6 *vs* 96.4 %). One PB AP case failed a mandatory constraint. Other dosimetric differences were non-significant. SIB AP plans took 14 min with one case failing a mandatory constraint with minor differences compared with MP except larger V _42.8Gy_ (3 *vs* 1.5 %) and more MU. VMAT AP plans took 12 min and were hotter for PTVp_4000 but had higher nodal coverage. Contra_Lung V _2.5Gy_ was higher (8.8 %) than MP plans (6.5 %).

**Conclusion:**

Automatic planning of modern breast techniques has been successfully introduced using a commercial planning system. AP plans are very similar to MP, requiring little manual interaction for most cases with significant timesaving potential.

**Advances in knowledge:**

Scripted breast plans produced within minutes for WB, PB, SIB and VMAT. Successfully introduced into large busy department. Plans similar to manual plans, requiring little manual interaction.

## Introduction

Breast cancer has now overtaken lung cancer as the world’s mostly commonly diagnosed cancer, according to statistics released by the International Agency for Research on Cancer (IARC) in 2020 with 2.3 million new cases.^1^ In females in the UK, breast cancer is the most common cancer, with around 56,000 new cases every year (2016–2018).^2^ Breast radiotherapy plays an important part of the multimodality treatment of breast cancer and a vital role in maximising local disease control, enabling safe breast conservation and contributing to increased survival.^3^ In our department, breast cancer has the highest referral for radiotherapy on average each month. Breast planning contributes significantly in terms of our workload with at least 1916 planning hours recorded in 2019. Breast radiotherapy planning has become increasingly personalised with techniques evolving in attempts to improve outcomes and minimise toxicity. We have seen shifts towards more hypofractionation whole breast (WB) and partial breast (PB) radiotherapy, use of simultaneous integrated boosts (SIB), complex volumetric modulated arc therapy (VMAT) or hybrid techniques as well as proton trials such as PARABLE.^4–7^

Automation is currently utilised in many steps in the radiotherapy pathway in departments. This can improve efficiency, reduce planning times and minimise errors while producing consistent high-quality clinical plans.^8^ There are a few approaches to automate planning depending on preference and tools, which can include scripting the steps (*e.g.* Raystation, Pinnacle^3^, and Eclipse), atlas knowledge-based planning (KBP) (*e.g.* RapidPlan module, Varian), deep learning (*e.g.* Raystation), iterative optimisation (Pinnacle Auto-Planning module, Philips Radiation ONCOLOGY Systems, Fitchburg, WI) and prior multicriteria optimisation (MCO, *e.g*. iCycle, Elekta). Most of these treatment planning systems (TPS) have some form of scripting environment to allow departments to customise the automation of planning steps to their local practice and procedures. The scripting approach allows good flexibility, is relatively easy to implement and does not require large libraries of data sets to build a model. In Raystation, there is currently a specific auto breast-planning module, which plans fully automatic tangential intensity modulated radiation therapy (IMRT) WB plans based on identifying surface placed wires.^9,10^ This was explored and can produce high quality clinically acceptable plans for most cases. For some, including our department, this may not always suit their local practice where multiple techniques need specific manual steps. Additionally, surface wires requires time and expertise during the CT scan. In our department, we have moved to 26 Gy in 5 fractions for whole and partial breast based on FAST FORWARD (FF) and IMPORT LOW.^4,5^ We also use a hybrid tangential and VMAT arc for SIB to 48 Gy in 15 fractions for those requiring a tumour boost following the protocol for IMPORT HIGH.^6^ Then, for nodal patients with internal mammary chain, we use VMAT planning, which has been shown to produce high quality plans achieving high coverage while minimising normal tissue doses.^10^,^11^

Although there is plenty in the literature demonstrating effectiveness of automated breast planning, the majority focuses on conventionally fractionated whole breast IMRT/VMAT and little explores tangential IMRT partial breast, hybrid SIB and VMAT for nodal patients all within the same script.^12–16^ These techniques and fractionations above are more relevant to current breast planning approaches, especially within the UK due to practice changing randomised controlled trials.^17^ This work describes the clinical implementation of script-based breast radiotherapy using the commercial Raystation TPS for a variety of modern techniques and compares against manual clinical plans within a busy NHS radiotherapy department.

## Methods and materials

### Cases

Ninety-three patients with breast cancer who were treated with manually created plans (MP) (32 WB/chest wall (CW), 20 PB, 21 SIB and 20 VMAT) were retrospectively re-planned using the scripted semi-automatic approach. Patients underwent either a planning deep inspiration breath hold (DIBH) or free breathing (FB) CT scan with 3 mm thick slices. All were positioned supine with their arms raised above their head on a breast board. See Table 1 for dose–volume constraints and organs at risk (OARs) used for planning. All plans were produced in Raystation V10A (Raysearch, Stockholm, Sweden) using a 2 mm dose grid and on either an Elekta Agility linear accelerator (Elekta Oncology Systems Ltd., Crawley, UK) or TruebeamStx (Varian Medical System, Palo Alto, CA). VMAT plans were planned only on Elekta. The same machine, dose algorithm and grid size was used when re-planning each case. The clinical manual plans were all produced by various competent staff members (Dosimetrists, Radiographers and Physicists) and checked before treatment. A single senior Dosimetrist produced the automatic scripted plans. The workflow for each technique is described below.

**Table 1.** Dose–volume constraints and clinical goals used for planning

Structure	Parameter	Objective	Constraint	Structure	Parameter	Objective	Constraint
***Whole / Partial breast***				***VMAT breast & nodes***			
**PTVp_2600**	V _24.7Gy_[%]	≥ 95 %	≥ 90 %	**PTVp_4000**	V _38Gy_ [%]	≥ 95 %	≥ 90 %
	V _27.3Gy_[%]	≤ 5 %	≤ 7 %		V _42.8Gy_ [%]		≤ 2 %
	V _27.82Gy_[%]		≤ 2 %		D _0.5cc_ [Gy]		≤ 44 Gy
	D _0.5cc_[Gy]		≤ 28.6 Gy	**PTV nodes**	V _36Gy_ [%]	≥ 90 %	
**Ipsi_Lung**	V _7.8Gy_[%]	≤ 15 %	≤ 17 %		V _32Gy_ [%]		≥ 80 %
**Heart (if outlined**)	V _6.5Gy_[%]		≤ 5 %		V _42.8Gy_ [%]		≤ 2 %
	V _1.3Gy_[%]		≤ 30 %		D _0.5cc_ [Gy]		≤ 44 Gy
**Body – PTV**	V _27.82Gy_[cc]	≤ 2 cc		**Ipsi_Lung**	V _17Gy_ [%]		≤ 35 %
	D _0.5cc_[Gy]		≤ 28.6 Gy		D _Mean_ [Gy]	≤ 13 Gy	
				**Contra_Lung**	V _2.5Gy_ [%]	≤ 3 %	
***SIB breast plans***					D _Mean_ [Gy]	≤ 1 Gy	
**PTVp_4800_DVH**	V _45.6Gy_[%]	≥ 95 %	≥ 90 %	**Heart**	V _13Gy_ [%]	≤ 2 %	≤ 10 %
	V _51.4Gy_[%]		≤ 2 %		D _Mean_ [Gy]	≤ 3 Gy	≤ 4 Gy
	D _0.5cc_[Gy]		≤ 52.8 Gy	**Contra_Breast**	D _Mean_ [Gy]		≤ 3.5 Gy
**PTVp_4000-PTVp_4800_DVH**	V _38Gy_[%]	≥ 95 %	≤ 90 %	**Oesophagus**	V _17Gy_ [%]	≤ 15 %	
	D _50%_ [Gy]	39–41.2 Gy			D _Mean_ [Gy]	≤ 11 Gy	
	V _48Gy_[%]	≤ 5 %		**Body – PTV**	V _42.8Gy_ [cc]	≤ 2 cc	
**PTVp_4000-PTVp_4800_DVH + 1** **cm**	V _42.8Gy_[%]	≤ 5 %			D _0.5cc_ [Gy]		≤ 44 Gy
	V _44Gy_[cc]	≤ 2 cc					
**Ipsi_Lung**	V _18Gy_[%]	≤ 10 %					
	D _Mean_ [Gy]	≤ 6 Gy					
**Contra_Lung**	V _2.5Gy_[%]	≤ 3 %	≤ 15 %				
	D _Mean_ [Gy]	≤ 1 Gy					
**Heart**	V _13Gy_[%]	≤ 2 %	≤ 10 %				
	D _Mean_ [Gy]	≤ 3 Gy	≤ 4 Gy				
**Contra_Breast**	D _Mean_ [Gy]	≤ 1 Gy	≤ 3.5 Gy				
**Body-PTVp_4000 + PTVp_4800_DVH + 1** **cm**	V _42.8Gy_[cc]	≤ 2 cc					
	D _0.5cc_[Gy]		≤ 44 Gy				

V _XGy_ = percentage of volume receiving XGy; D _0.5cc_ = Dose to 0.5 cc volume in Gy; Ipsi = ipsilateral; Contra = contralateral; D _Mean_ = mean dose; Body-PTV = body outside PTV.

### Tangential field placement (whole breast, partial breast, hybrid SIB plans only)

For plans having tangential IMRT fields (WB, PB, hybrid SIB), a senior Dosimetrist/Radiographer placed a medial tangential beam manually to cover the WB/chest wall tissue with a 1 cm margin. A larger 2 cm anterior flash margin is applied. An attempt is made to keep maximum lung depth ≤15 mm, maximum liver depth ≤10 mm and maximum heart depth ≤10 mm. Any tumour bed clips should be ≥18 mm from the posterior edge of the field and the field must not ideally cross ≥2 cm over midline. If any of these guidelines are not possible, then some compromise is made and the Oncologist will review, make any required changes to the beam and approve for treatment. Otherwise, a second senior Dosimetrist/Radiographer can perform the second check/approval. Heart shielding using multileaf collimators (MLCs) for upper quadrant tumours is sometimes required, as long as there is adequate coverage of the tumour bed. DIBH for left-sided lower quadrant tumours or chest walls is frequently used.

### Whole breast tangential IMRT plans [26 Gy/5 fractions, 32 cases (15 right/17 left)]

These plans started with the approved medial field as described above. Plans consisted of a step and shoot IMRT technique (SMLC) of opposed tangential fields to treat the breast/chest wall. The posterior border of the tangents are aligned and additional tangential segments (~4–8) added by inverse optimisation to produce a homogenous dose distribution. The plan has two main “open” tangent beams (6 or 10 MV) which deliver approximately 75% of the dose with the remainder by the segments (6 MV). The open tangential beams have a flash margin of 2 cm from the body while the segments did not require such. The max number of segments was mostly set at 8, the minimum segment area set at 9 cm² and the minimum monitor units (MU) was 5. The WB/chest wall planning target volume (PTVp-2600) was a field-based volume based on the tangential fields. This was clipped away from the body and ipsilateral lung (Ipsi_Lung) by 5 mm and the prescription dose was 26 Gy in 5 fractions to the median of PTVp_2600. This approach has been described in the FF trial planning pack.^4^ These WB manual plans were checked and used for treatment. The Ipsi_Lung contour was created using multiatlas-based segmentation (MBS) and edited if needed by the planner.

### Partial breast tangential IMRT plans [26 Gy/5 fractions, 20 cases (12 right/8 left)]

These SMLC plans followed the same WB steps above with the following exceptions. The Oncologist outlined the heart and CTVp_Partialas per IMPORT LOW trial and planner grew this by 15 mm to a “PTV guide” contour.^5^ The tangential field lengths were then modified to align to this “PTV guide”. This new shortened field was then used to create the field-based PTVp_2600 similar to the WB method (clipping from body and Ipsi_Lung by 5 mm).

### SIB hybrid plans [48 Gy/15 fractions, 21 cases (7 right/14 left)]

The Oncologist outlined the CTV_TB and heart as per the IMPORT HIGH planning pack and the planner outlined the contralateral breast.^6^ The PTV_TB was created by growing the CTV_TB by 5 mm. These plans then followed the WB steps above, optimising the tangential IMRT WB fields to 40 Gy in 15 fractions. A single 6 MV partial VMAT beam was then applied to deliver the remaining 8 Gy in 15 fractions to give 48 Gy in 15 fractions to the median of PTV_TB_DVH (PTV_TB cropped 5 mm below skin surface). This VMAT beam rotated from the medial tangent gantry angle to the lateral tangent gantry angle with a collimator of 10°. The background dose of 40 Gy from the WB tangents was taken into consideration while optimising the VMAT arc. The arc field only conformed to the PTV_TB and planning bolus was used if needed for optimisation and removed for final dose calculation/normalisation. Optimisation ring structures were often added to aid planning conformity.

### VMAT nodal plans [40 Gy/15 fractions, 20 cases (6 right/14 left)]

These plans did not require any medial tangential field applied initially. Outlining of the nodal CTVs and OARs were done manually by the Oncologist as per ESTRO guidelines. The contralateral breast was outlined by the planner.^18^ All CTVs were grown by 5 mm to create their respective PTV volumes. These plans consisted of a 6 MV dual arc rotating from gantry angle 310° to 179° for left-sided and 50° to 181° for right-sided. The VMAT maximum dose rate was 550 MU/min with MLC leaf width of 5 mm, a constrained leaf motion of 0.6 cm/degree and gantry angle spacing of 4°. Collimator angles were rotated by 10° and reversed for the second arc. Each VMAT plan consisted of the first arc rotating from medial to lateral and then the second opposite direction. PTV coverage was priority and only compromised to achieve OAR mandatory constraints. The VMAT plans optimised to the PTVp_4000, which was clipped 5 mm from body. Plans had skin flash, achieved by simulated organ motion robust optimisation. This method has been previously described.^19^ Optimisation rings around the PTV targets and dose fall-off objectives were used frequently in an attempt to conform the dose and minimise hot spots.

### Scripting automatic plans

**Figure 1.**
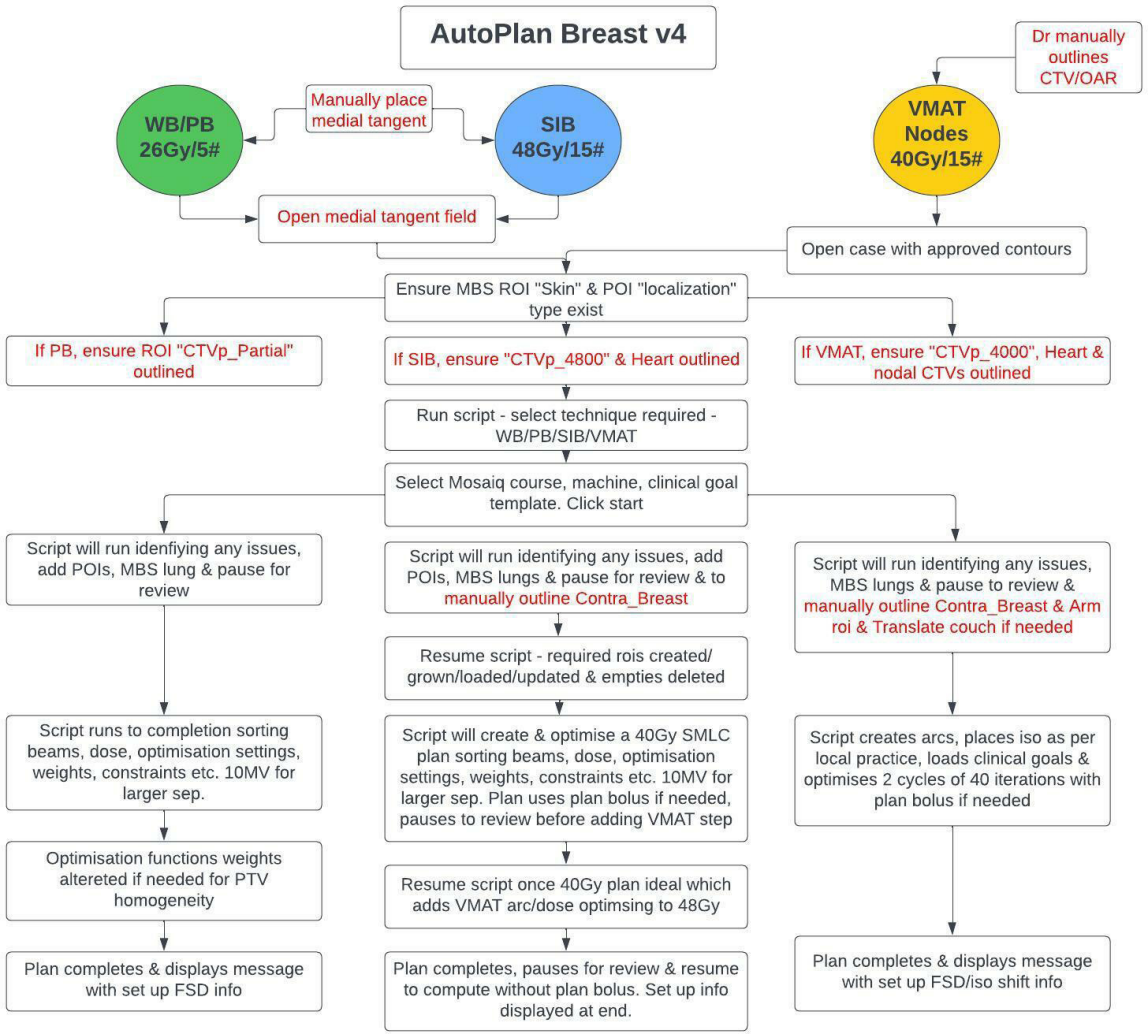
Scripted breast planning workflow. Manual steps are in red. CTV, clinical target volume; MBS, multiatlas-based segmentation; OAR, organ at risk; PB, partial breast; POIs, points of interest; PTV, planning target volume; ROI, region of interest; SIB, simultaneous integrated boost; VMAT, volumetric arc therapy; WB, whole breast.

A senior Dosimetrist wrote the code in Python language (v. 3.6) for Raystation scripting environment and the workflow for each technique is shown in Figure 1. The script aimed to complete as many of the manual steps as possible as described above. In addition to the steps in Figure 1, the script also creates tattoo points of interests (POIs), beams and prescriptions, aligns lateral tangent gantry angle, sets askin flash margin of 2 cm on tangential beams, uses 10 MV where separation is >27 cm, sets optimisation settings, loads clinical goals and creates a plan. Patient specific MLC treat margins are also used for tangents depending on chest wall thickness. For VMAT plans, isocenter placement is based on local collision avoidance criteria and cone beam CT (CBCT) imaging. The scripted plans were timed with a stopwatch from starting until plan completion. The time excluded any time that would be usually required to outline any manual contours needed, such as the contralateral breast in SIB/VMAT plans, an arm avoidance contour in VMAT plans and translating of the couch contour in VMAT plans if necessary. AP plans did not have any human manual adjustments once the script completed, except when it paused to ensure the MBS lung structure/s created were acceptable (which in most cases requiredno editing). To evaluate potential time saving, the current allocated manual planning time was reviewed against the time taken to perform the automatic plans.

### Statistical analyses

Plan comparisons and statistical analysis were carried out between the techniques using IBM SPSS statistical package. Plans were compared using two-tailed Wilcoxon signed rank tests with *p*-values ≤ 0.05 indicating statistical significance.

## Results

### Whole breast tangential IMRT plans

**Figure 2.**
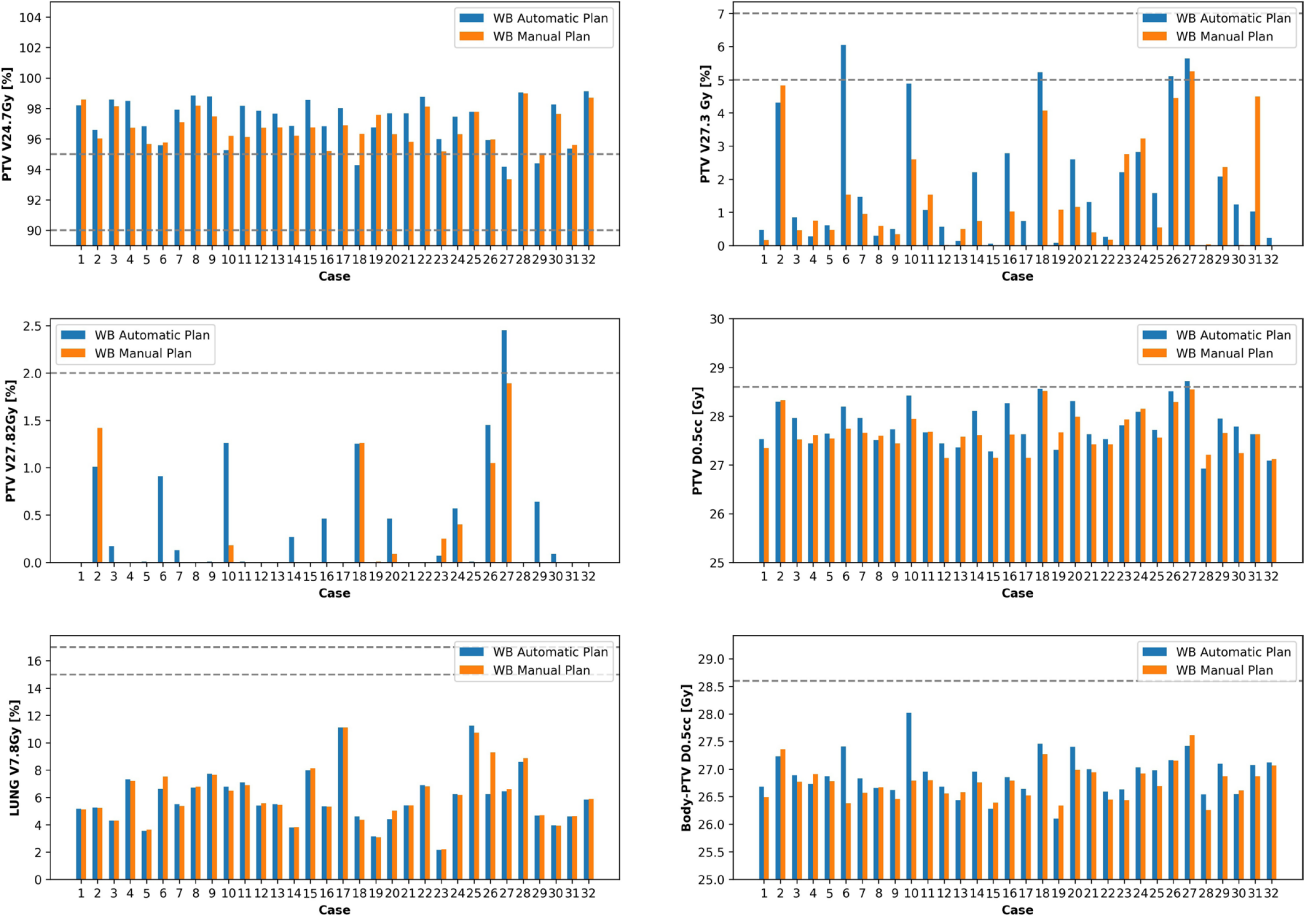
Scripted automatic and manual plans for whole breast 26 Gy/5 fractions. Dashed lines represent dose constraints from Table 1. Where data are missing, it is because the isodose did not occur on the plan. WB, whole breast.

Table 2 shows that the automatic plans achieved very similar dose statistics to manually created plans with the scripted plans completing within 7 min. Mean PTVp_2600 V _24.7Gy_ was 96.7 and 97.2% for MP and AP, respectively. There were statistically significant differences with the AP plans being slightly hotter and with higher coverage. Most differences were <1% for V _XGy_ and <0.2 Gy for D _0.5cc_. Figure 2 shows each individual case dose comparison. All manual plans met constraints while only one AP case (case 27) failed to meet a dose constraint and for this plan had a V _27.82Gy_ of 2.45% and a D _0.5cc_ of 28.72 Gy. For this case, the MP had a V _24.7Gy_of 93.3% slightly lower than the AP 94.2%. Five AP cases failed 1–2 dose objective while only one case for the MP plans.

**Table 2.** Plan dose statistics for whole breast and partial breast tangent-based IMRT manually created clinical plans and scripted automatic plans

Structure	Parameter	Manual		Auto plan			
***Whole breast plans [n = 32]***		***Mean ± SD***	***Range***	***Mean ± SD***	***Range***	**Mean difference**	*p*-value
**PTVp_2600**	V _24.7Gy_[%]	96.7 ± 1.2	93.3–99.0	97.2 ± 1.5	94.2–99.1	0.5 %	**0.0016**
	V _27.3Gy_[%]	1.5 ± 1.6	0–5.3	1.8 ± 1.8	0–6.1	0.3 %	**0.0455**
	V _27.82Gy_[%]	0.2 ± 0.5	0–1.9	0.4 ± 0.6	0–2.5	0.2 %	**0.0071**
	D _0.5cc_[Gy]	27.7 ± 0.4	27.1–28.6	27.8 ± 0.4	26.9–28.7	0.1 Gy	**0.0031**
**Ipsi_Lung**	V _7.8Gy_[%]	6.0 ± 2.0	2.2–11.1	5.9 ± 2.0	2.2–11.2	0.1 Gy	NS
**Body – PTV**	V _27.82Gy_[cc]	0.007 ± 0.02	0–0.15	0.05 ± 0.2	0–1.3	0.043 cc	NS
	D _0.5cc_[Gy]	26.8 ± 0.3	26.3–27.6	26.9 ± 0.4	26.1–28.0	0.1 Gy	**0.0032**
**MUs**		604.5 ± 28.7	554.4–648.4	613.9 ± 25.2	561.5–643.4	9.4 μ	**0.0043**
**Time**	Minutes			4.8 ± 0.8	3.5–6.2		
***Partial breast plans [ n = 20]***		***Mean ± SD***	***Range***	***Mean ± SD***	***Range***	**Mean Difference**	*p*-value
**PTVp_2600**	V _24.7Gy_[%]	96.4 ± 2.2	90.6–98.9	97.6 ± 1.8	92.9–99.4	1.2 %	**<0.001**
	V _27.3Gy_[%]	1.5 ± 2.0	0–5.3	1.8 ± 2.4	0–7.5	0.3 %	NS
	V _27.82Gy_[%]	0.3 ± 0.5	0–1.6	0.4 ± 0.9	0–3.6	0.1 %	NS
	D _0.5cc_[Gy]	27.5 ± 0.5	26.9–28.4	27.5 ± 0.7	26.6–28.9	0 Gy	NS
**Ipsi_Lung**	V _7.8Gy_[%]	4.6 ± 2.0	2.2–9.4	4.7 ± 2.1	2.3–10.7	0.1 %	NS
**Heart**	V _1.3Gy_[%]	1.3 ± 2.9	0–11.0	1.5 ± 3.3	0–13.1	0.5 %	NS
	V _6.5Gy_[%]	0 ± 0	0–0	0.003 ± 0.01	0–0.06	0.003 %	NS
**Body – PTV**	V _27.82Gy_[cc]	0.017 ± 0.06	0–0.3	0.08 ± 0.18	0–0.7	0.063 cc	NS
	D _0.5cc_[Gy]	26.8 ± 0.4	26.2–27.7	26.7 ± 0.6	26.6–28.9	0.1 Gy	NS
**MUs**		623.8 ± 30.3	572.7–693.2	624.3 ± 24.4	586.5–661.7	0.5 MU	NS
**Time**	Minutes			6.4 ± 1.1	4.8–8.8		

V _XGy_ = percentage of volume receiving XGy; D _0.5cc_ = Dose to 0.5 cc volume in Gy; Ipsi = ipsilateral; Contra = contralateral; D _Mean_ = mean dose; Body-PTV = body outside PTV; MUs = monitor units; NS = not significant. *p*-values ≤ 0.05 are shown in bold.

### Partial breast tangential IMRT plans

**Figure 3.**
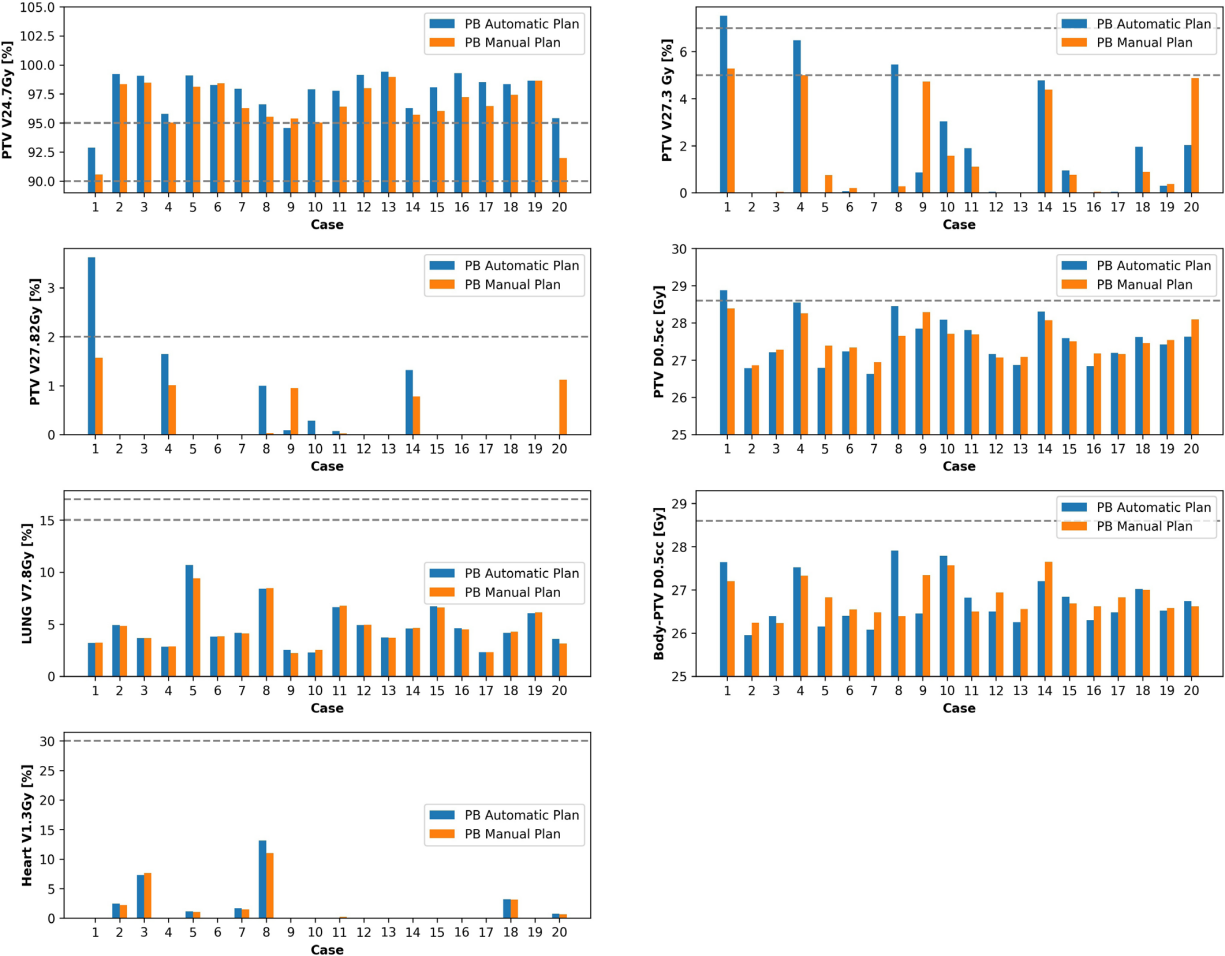
Scripted automatic and manual plans for partial breast 26 Gy/5 fractions. Dashed lines represent dose constraints from Table 1. Where data are missing, it is because the isodose did not occur on the plan. PB, partial breast; PTV, planning target volume.

Only the PTV_2600 V _24.7Gy_ in Table 2 was statistically significantly different but this was marginal with a mean difference of 1.2% (96.4% MP and 97.6% AP) in favour of the AP. Table 2 shows that the automatic plans achieved very similar dose statistics to manually created plans and completed within 9 min. Figure 3 shows that no MP failed any constraints while only one AP case did (case 1 V _27.82Gy_ 3.62 %, V _27.3Gy_ 7.5% and D _0.5cc_28.9 Gy. For this case, the MP had a lower V _24.7Gy_ of 90.6 *vs* 92.9 % for the AP. Four cases failed at least one dose objective for the AP plans (case 1, 4, 8 and 9) and three cases for the MP plans (case 1, 4 and 20).

### SIB hybrid plans

**Figure 4.**
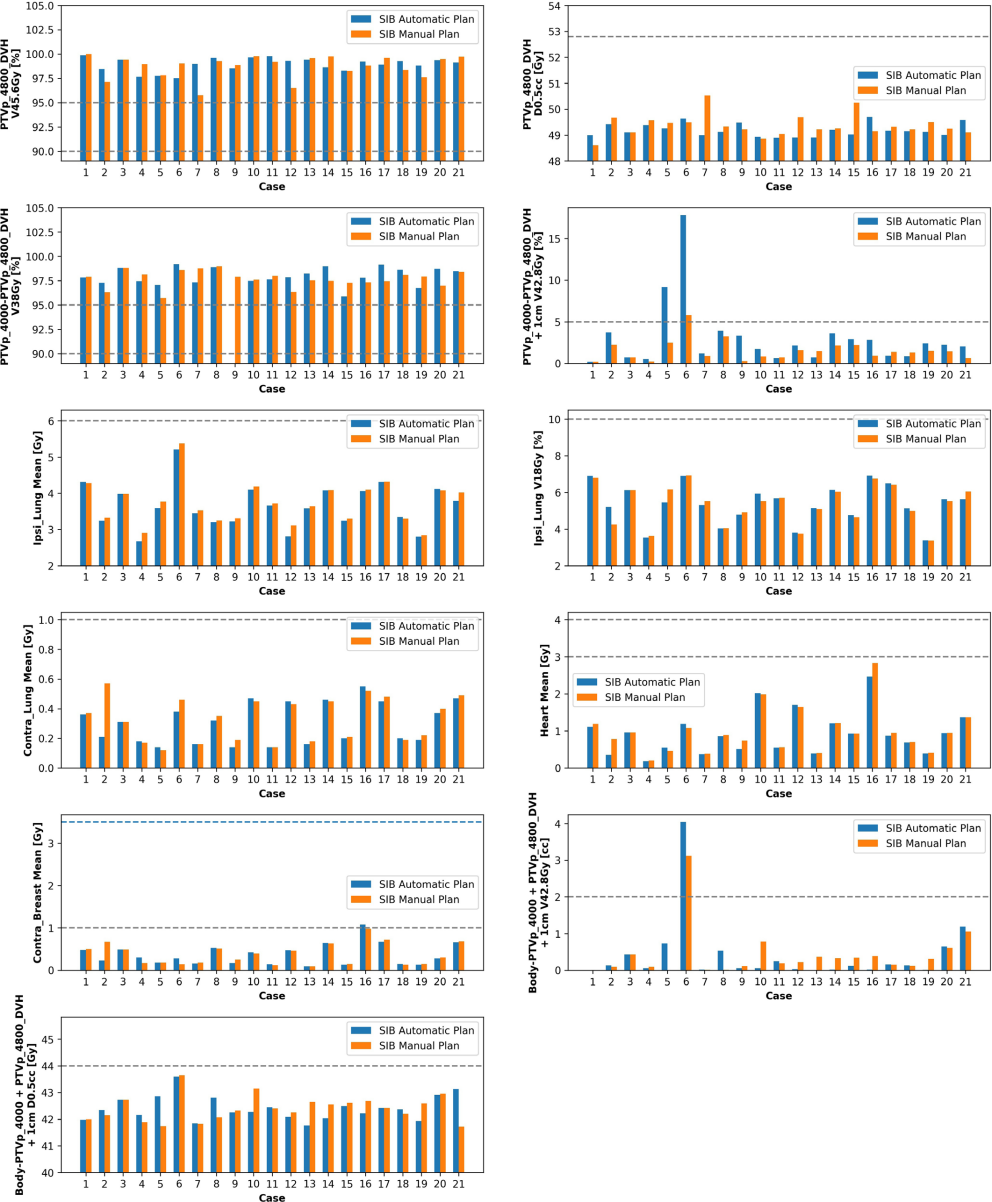
Scripted automatic and manual plans for SIB breast 48 Gy/15 fractions. Dashed lines represent dose constraints from Table 1. Where data are missing, it is because the isodose did not occur on the plan. SIB, simultaneous integrated boost; PTV, planning target volume.

Table 3 shows a similar high level of target coverage for both AP and MP plans for whole breast and tumour bed. AP plans were completed within 14 min. PTVp_4000-PTVp_4800_DVH + 1 cm V _42.8cc_ had a slightly higher value of 3 *vs* 1.5 % in favour of MP. The MUs were also slightly larger for the AP plans (mean increase of 14 MU). Figure 4 shows that only one AP plan failed a constraint (case 9 **-** PTVp_4000-PTVp_4800_DVHV _38Gy_ 84.5 %). Three cases failed at least one dose objective for the AP plans (case 5, 6 and 16) and one case in the MP (case 6).

**Table 3.** Plan dose statistics for hybrid tangent whole breast with VMAT SIB manually created clinical plans and scripted automatic plans

Structure	Parameter	Manual		Auto Plan			
***SIB breast plans [n = 21]***		***Mean ± SD***	***Range***	***Mean ± SD***	***Range***	**Mean Difference**	*p*-value
**PTVp_4800_DVH**	V _45.6Gy_[%]	98.7 ± 1.2	95.8–99.99	98.9 ± 0.7	97.5–99.9	0.2 %	NS
	V _51.4Gy_[%]	0 ± 0	0–0	0 ± 0	0–0	0 %	NS
	D _0.5cc_[Gy]	49.4 ± 0.4	48.6–50.5	49.2 ± 0.3	48.9–49.7	0.2 Gy	NS
**PTVp_4000-PTVp_4800_DVH**	V _38Gy_[%]	97.7 ± 0.9	95.7–98.9	97.3 ± 3.1	84.5–99.2	0.4 %	NS
	D _50%_ [Gy]	40.5 ± 0.3	40.2–41.4	40.5 ± 0.3	40.3–41.8	0 Gy	NS
	V _48Gy_[%]	0.02 ± 0.06	0–0.32	0.003 ± 0.01	0–0.05	0.017 %	NS
**PTVp_4000-PTVp_4800_DVH + 1** **cm**	V _42.8Gy_[%]	1.5 ± 1.3	0.2–5.8	3.0 ± 3.9	0.2–17.8	1.5 cc	**0.0025**
	V _44Gy_[cc]	0.4 ± 0.7	0–2.6	0.3 ± 0.9	0–4.1	0.1 cc	NS
**Ipsi_Lung**	V _18Gy_[%]	5.3 ± 1.1	3.4–6.9	5.4 ± 1.1	3.4–6.9	0.1 %	NS
	D _Mean_ [Gy]	3.7 ± 0.6	2.8–5.4	3.7 ± 0.6	2.7–5.2	0 Gy	**<0.001**
**Contra_Lung**	V _2.5Gy_[%]	0.01 ± 0.06	0–0.31	0.03 ± 0.1	0–0.55	0.02 %	NS
	D _Mean_ [Gy]	0.3 ± 0.1	0.12–0.6	0.3 ± 0.1	0.14–0.6	0 Gy	NS
**Heart**	V _13Gy_[%]	0.07 ± 0.3	0–1.3	0.06 ± 0.2	0–1.0	0.01 %	NS
	D _Mean_ [Gy]	0.9 ± 0.6	0.2–2.8	0.9 ± 0.6	0.2–2.5	0 Gy	NS
**Contra_Breast**	D _Mean_ [Gy]	0.4 ± 0.3	0.09–0.9	0.4 ± 0.3	0.1–1.1	0 Gy	NS
**Body-PTVp_4000 + PTVp_4800_DVH + 1** **cm**	V _42.8Gy_[cc]	0.4 ± 0.7	0–3.1	0.4 ± 0.9	0–4.0	0 cc	NS
	D _0.5cc_[Gy]	42.4 ± 0.5	41.7–43.7	42.4 ± 0.5	41.8–43.6	0 Gy	NS
**MUs**		395.4 ± 15.2	365.9–439.2	409.4 ± 20.3	386.2–460.3	14 MU	**0.0019**
**Time**	Minutes			10.5 ± 2.0	6.4–13.4		

V _XGy_ = percentage of volume receiving XGy; D _0.5cc_ = Dose to 0.5 cc volume in Gy; Ipsi = ipsilateral; Contra = contralateral; D _Mean_ = mean dose; Body-PTV = body outside PTV; MUs = monitor units; NS = not significant. *p*-values ≤ 0.05 are shown in bold.SIB, simultaneous integrated boost; VMAT, volumetric arc therapy.

### VMAT nodal plans

**Figure 5.**
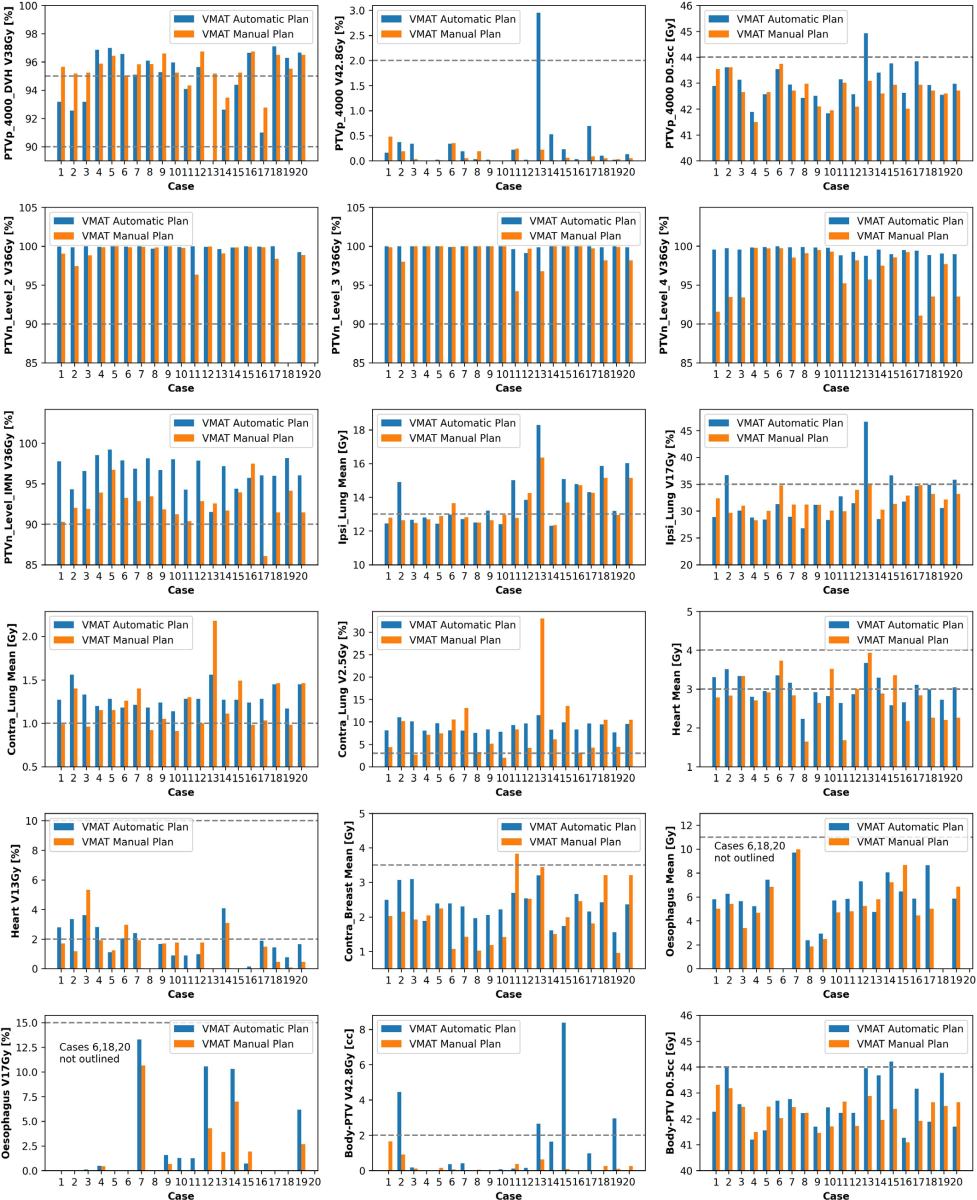
Scripted automatic and manual VMAT plans for nodal patients 40 Gy/15 fractions. Dashed lines represent dose constraints from Table 1. Where data are missing, it is because the isodose did not occur on the plan. PTV, planning target volume; VMAT, volumetric arc therapy.

VMAT AP plans were produced within 12 min. Table 4 shows small differences between the plans with the most obvious difference being higher coverage of nodal targets for AP plans. The Contra_Lung V _2.5Gy_ was higher for the AP plans with a mean of 8.8 *vs* 6.5 %. Figure 5 shows that four AP cases failed at least one dose constraint (case 2, 13, 15 and 20) and two MP cases (case 11 and 17). Regarding dose objectives, AP failed more than the MP (78 *vs* 51) over all cases, with the most commonly failed objectives being Contra_Lung V _2.5Gy_, Contra_Lung D _Mean_ and Ipsi_Lung D _Mean_.

**Table 4.** Plan dose statistics for whole breast and nodes (including IMN) with VMAT manually created clinical plans and scripted automatic plans

Structure	Parameter	Manual		Auto plan			
***VMAT breast & nodes plans [n = 20]***		***Mean* ± *SD***	***Range***	***Mean* ± *SD***	***Range***	**Mean difference**	*p*-value
**PTVp_4000**	V _38Gy_ [%]	95.5 ± 1.1	92.8–96.7	94.6 ± 2.9	85.0–97.1	0.9 %	NS
	V _42.8Gy_ [%]	0.1 ± 0.13	0–0.48	0.3 ± 0.6	0–3.0	0.2 %	**0.0278**
	D _0.5cc_ [Gy]	42.7 ± 0.6	41.5–43.7	43.0 ± 0.7	41.8–44.9	0.3 Gy	**0.0244**
**PTVn_Level_1**	V _36Gy_ [%]	99.2 ± 1.1	96.7–100	99.9 ± 0.1	99.8–100	0.7 %	**<0.001**
	V _42.8Gy_ [%]	0.002 ± 0.006	0–0.02	0.01 ± 0.06	0–0.21	0.008 %	NS
	D _0.5cc_ [Gy]	41.1 ± 0.4	40.6–42.0	41.3 ± 0.5	40.7–42.4	0.2 Gy	NS
**PTVn_Level_2**	V _36Gy_ [%]	99.3 ± 1.0	96.4–100	99.9 ± 0.2	99.3–100	0.6 %	**0.0031**
	V _42.8Gy_ [%]	0.01 ± 0.04	0–0.19	0.002 ± 0.009	0–0.04	0.008 %	NS
	D _0.5cc_ [Gy]	41.5 ± 0.5	40.6–42.5	41.4 ± 0.4	41–42.5	0.1 Gy	NS
**PTVn_Level_3**	V _36Gy_ [%]	99.2 ± 1.5	94.2–100	99.9 ± 0.2	99.1–100	0.1 %	**0.0124**
	V _42.8Gy_ [%]	0.13 ± 0.42	0–1.45	0.015 ± 0.05	0–0.18	0.115 %	NS
	D _0.5cc_ [Gy]	41.4 ± 0.8	40.0–43.0	41.3 ± 0.4	40.8–42.6	0.1 Gy	NS
**PTVn_Level_4**	V _36Gy_ [%]	96.7 ± 3.0	91.0–99.8	99.4 ± 0.4	98.7–99.9	2.7 %	**<0.001**
	V _42.8Gy_ [%]	0.05 ± 0.14	0–0.57	0.05 ± 0.14	0–0.61	0 %	NS
	D _0.5cc_ [Gy]	41.7 ± 0.5	40.5–42.7	41.6 ± 0.4	41.2–42.7	0.1 Gy	NS
**PTVn_Level_Interpect**	V _36Gy_ [%]	99.9 ± 0.06	99.9–100	99.9 ± 0.1	99.8–100	0 %	N/A
	V _42.8Gy_ [%]	0 ± 0	0–0	0.03 ± 0.065	0–0.13	0.03 %	N/A
	D _0.5cc_ [Gy]	41.1 ± 0.6	40.5–41.6	41.3 ± 0.6	40.8–42.2	0.2 Gy	N/A
**PTVn_Level_IMN**	V _36Gy_ [%]	92.5 ± 2.4	86.1–97.5	96.5 ± 1.9	91.5–99.2	4.0 %	**<0.001**
	V _42.8Gy_ [%]	0.09 ± 0.2	0–0.8	0.3 ± 0.5	0–1.6	0.21 %	NS
	D _0.5cc_ [Gy]	41.7 ± 0.5	40.6–42.5	42.2 ± 0.4	41.3–42.9	0.5 Gy	**0.0016**
**Ipsi_Lung**	V _17Gy_ [%]	31.8 ± 1.9	28.3–35.0	32.1 ± 4.5	26.8–46.6	0.3 %	NS
	D _Mean_ [Gy]	13.5 ± 1.1	12.4–16.4	13.9 ± 1.6	12.3–18.3	0.4 Gy	NS
**Contra_Lung**	V _2.5Gy_ [%]	6.5 ± 3.3	2.0–13.1	8.8 ± 1.0	7.5–11.0	2.5 %	**0.0131**
	D _Mean_ [Gy]	1.2 ± 0.3	0.9–2.2	1.2 ± 0.1	1.1–1.6	0 Gy	NS
**Heart**	V _13Gy_ [%]	1.5 ± 1.3	0–5.3	1.8 ± 1.2	0–4.0	0.3 %	NS
	D _Mean_ [Gy]	2.8 ± 0.6	1.6–3.9	3.0 ± 0.4	2.2–3.7	0.2 Gy	NS
**Contra_Breast**	D _Mean_ [Gy]	2.1 ± 0.8	1.0–3.8	2.3 ± 0.5	1.6–3.2	0.2 Gy	NS
**Oesophagus**	V _17Gy_ [%]	1.7 ± 3.0	0–10.1	2.7 ± 4.4	0–13.3	1.0 %	NS
	D _Mean_ [Gy]	5.4 ± 2.0	1.9–10.0	6.1 ± 1.8	2.4–9.7	0.7 Gy	NS
**Body-PTV**	V _42.8Gy_ [cc]	0.2 ± 0.4	0–1.7	1.1 ± 2.1	0–8.4	0.9 %	NS
	D _0.5cc_ [Gy]	42.2 ± 0.6	41.1–43.3	42.2 ± 0.9	41.2–44.2	0 Gy	NS
**MUs**		597.6 ± 139.7	439.3–1041.2	584.3 ± 85.0	489.3–842.96	13.3 MU	NS
**Time**	Minutes			9.5 ± 0.7	8.4–11.4		

Body-PTV, body outside PTV; Contra, contralateral; D Mean, mean dose; D 0.5cc, Dose to 0.5 cc volume in Gy; Ipsi, ipsilateral; MUs, monitor units; NS, not significant; VMAT, volumetric arc therapy;VXGy, percentage of volume receiving XGy.

p-values ≤ 0.05 are shown in bold

### Clinical impact

The breast script has been introduced into our clinic currently for WB and PB and is undergoing further final validation for the SIB and VMAT, which will be released imminently. A local scripting team has been set up in our department to manage scripts in a safe and effective manner. They meet monthly to ensure certain scripts are required, fit for purpose, tested and released appropriately. Feedback is collected continuously from staff and used to improve future versions. The potential for time savings is shown in Table 5, where it reports the total planning time recorded for the year 2019 (WB/PB/SIB) and 2021 (VMAT). The current default times allocated to planning does include administration time such as checklists, printing skin rendered images, reviewing the plan and outlining any required contours. Based on the scripting times and results in this work, it is of the authors opinion that it would not be unreasonable to expect a reduction in the times to 0.75, 2 and 4 h for WB/PB, SIB and VMAT plans which could lead to a potential time saving of 848 planning hours per year for our department.

**Table 5.** Estimated potential time savings to department from using the scripted automatic planning approach

Plan type	Planning hours recorded	Default manual hours per plan	Est.AutoPlan hours per plan	Hours saved per plan	Percentage change	Hours saved per year
						
**WB/PB plan 2019**	979.8	1.75	0.75	1	57.1%	559.9
**SIB plan 2019**	545.1	3.5	2	1.5	42.9%	233.6
**VMAT plan 2021**	164.5	6	4	2	33.3%	54.8
**Total**	1689.4	11.25	6.75	4.5	40 %	848.3

PB, partial breast; SIB, simultaneous integrated boost; VMAT, volumetric arc therapy; WB, whole breast.

## Discussion

The aim of this study was to introduce into the clinic semi-automatic scripted breast planning for a range of current personalised techniques. To our knowledge, this is the first study that, under one tool, automates WB, PB, SIB and nodal VMAT using recent high quality trial techniques.

### Whole breast tangential IMRT plans [26 Gy/5 fractions, 32 cases]

For the WB cases, all but one (case 27) passed the mandatory dose constraints without further manual planning. This case 27 was a challenging one as the clinical MP also failed some objectives showing that some additional planning effort was required. Therefore, ~97 % cases are deemed clinically acceptable, producing a plan within 7 min. Similar times of 5–7 min have been also found for auto planning WB tangential IMRT.^14,15,20^ The high acceptance rate of ~97% for our WB plans match closely to those found by Purdie et al who validated prospectively over 1661 cases, the use of fully automatic planning for tangential IMRT using segmentation and planning.^10^ Here, the breast target was created automatically with the aid of radiopaque wires on the skin placed at CT. The beams were created automatically with user input preferences for OAR /target. In our study, we choose to keep the initial medial tangent field placement a manual process as we feel it requires a more personalised approach. It also does not require additional expertise and time at CT to place wires on the skin.

### Partial breast tangential IMRT plans [26 Gy/5 fractions, 20 cases]

Only one case (case 1) from 20 PB AP plans failed a dose constraint resulting in an acceptable clinical plan rate of 95% completing within 9 min. This case 1 was slightly more complicated and required further optimisation as shown by the fact the manual clinical plan still did not pass the objective for PTV V _24.7Gy_ which was 90.6% and PTV V _27.3Gy_. There is little in the literature regarding automation of this PB technique, which is a based on “mini tangents“, as used in the IMPORT LOW breast trial test arm 2.^5^ The trial has reported non-inferiority of mini tangential PB RT compared to standard WB RT using 40 Gy in 15 fractions. For our WB patients we give 26 Gy in 5 fractions based on the FF trial and as per COVID RCR guidelines have moved to give this fractionation also for PB patients.^21^ IMPORT LOW has the same control group fractionation as FF. Another paper also looked at hypofractionation automation of PB planning but using VMAT.^22^ Here, Marrazzo et al compared AP in Pinnacle *vs* manual planning for 30 Gy/5 fractions and found AP plans to be at least as good as MP and with lower MU and planning time under 10 min. Our time found was similar (up to 9 min). The additional time compared to WB is for the creating of “PTV guide” contour to help create the shortened tangential fields and then checking for possible machine violations of jaws/MLCs. As more departments adopt this PB technique which is geared toward early stage low risk patients, this technique will used more often clinically.

### SIB hybrid plans [48 Gy/15 fractions, 21 cases]

Only one SIB AP plan case (case 6) from 21 failed a mandatory dose constraint giving a clinically acceptable plan rate of 95.2% and completed within 14 min. The SIB technique, based on the IMPORT HIGH trial (48 Gy to tumour bed) is expected to expand to more centres based on recommendations in the UK. The sequential boost delivered in the control arm was not advantageous in terms of toxicity/local control compared to when the boost is delivered as a SIB; and in terms of TB dose escalation (53 Gy in 15 fractions to the TB PTV), this has not shown much benefit in terms of local disease controlbut reports a slightly higher risk of breast toxicity (at 5 years). Hence, 48 Gy in 15 fractions to the TB PTV delivered as a SIB has started to be introduced in centres, but no dose reduction yet to the WB PTV (as per the test arms of IMPORT HIGH) as the later follow-up data are awaited.^6,17,23^ The advantages of these techniques (WB, PB, and SIB) are that they predominantly employed simple tangential fields making the technique implementable in centres worldwide. The amount of MU was statistically significantly larger for the AP plans for WB plans (mean increase ~9.4 MU) and SIB plans (mean increase ~14 MU). These changes are relatively small in relation to the total plans MU and therefore not considered to be of clinical significance. Possible reasons for the increase may include the increased coverage/hotness on WB AP plans. Additionally, the SIB AP plans used a 5 mm MLC margin around the PTVTB for the VMAT beam and the planner of the manual plans did not always do this.

### VMAT nodal plans [40 Gy/15 fractions, 20 cases]

The VMAT nodal plans were able to automate most of the workflow apart from initial CTV/OAR outlining. The plans produced gave reasonable dosimetry requiring little additional manual interaction by the planner. The higher Contra_Lung lower doses for AP plans may need further attention to reduce more in a future version of the script. The objective of V _2.5Gy_ is quite strict and is the most often one not met in manual plans (only 3/20 cases met this). The clinical significance of this is likely to be small as this objective is not routinely used in the literature and more of an optimisation goal to avoid low dose spillage. The AP plans has also reduced errors associated with isocenter placement, which in turn reduces risks of re-planning and collisions during treatment with patient/equipment. Cilla et al looked at automated Pinnacle VMAT planning for chest wall and nodes and found plans were produced under 30 min with lower OAR doses.^24^ Van Duren-Koopman used hybrid tangents with superior nodal VMAT scripting in Eclipse TPS to produce comparable plans with only 5 min interaction time.^25^ Our work provides us with the ability to produce near finished plans with minimal manual effort. This has become increasingly important for proton trials where often patient eligibility is based on evaluating the photon plan prior to trial inclusion. Producing these plans more efficiently can therefore help streamline this trial process.

For the WB plans, the AP plans were slightly hotter than MP and in some cases failed a dose constraint. 4/32 cases failed V_27.3Gy_ objective (case max increase 4.5 %), one case failed V_27.8Gy_ mandatory (increase 0.56 %) and one case failed D_0.5cc_ mandatory (increase 0.17 Gy). For PB plans, the AP plans were slightly hotter on occasion than MP (not statistically significantly) with some cases failing a dose constraint. 3/20 cases failed V_27.3Gy_ objective (max increase 5.2 %), one case failed V_27.8Gy_ mandatory (increase 2.0 %) and one case failed D_0.5cc_ mandatory (increase 0.49 Gy).

For SIB plans, the AP plans were slightly hotter on occasion than MP with 2/21 cases failing the V_42.8Gy_ objective (max increase 12 %), one case failed the V_44Gy_ objective (increase 1.5 %). These objectives are in-house based on local experience, so it is difficult to interpret any clinical effect. The IMPORT HIGH trial looked at the prescribed dose spillage outside PTV_TB (constraint <5% control arm) which in this study, it is comforting to note it was <1% for both AP and MP plans.

For VMAT plans, the AP plans were slightly hotter on occasion than MP with one case failing the V_42.8Gy_ objective (increase 2.7 %) and one case failed D_0.5cc_ mandatory (increase 1.8 Gy). Overall, the majority of plans met the mandatory constraints being clinically acceptable. On the odd occasion, slight manual editing of MLC is likely needed to shield a hot region to ensure the plan meets mandatory constraints. This manual editing would unlikely be a lengthy process and probably take a few minutes. The magnitude of any dose increase between MP and AP plans appear reasonably small and not expected to translate to clinically relevant breast toxicity differences. Future development of the script aims to improve plan quality and explore additional features such as multicrieria optimisation and a new feature in Raystation 12A called “fine tune optimisation”.

This study is limited by a relatively small sample especially for the PB, SIB and VMAT plans. Despite this, it is evident that the AP plans dose distributions are quite similar to the MP plans with great time-saving potential. The outlining of the lung contours was done using MBS for the manual plans and then was repeated in the AP plans by the script. For both plans, the planner reviewed the contours and made any required edits, so it is possible that the contours differed slightly. However, both MP and AP plans used contours that would be acceptable for clinical purposes and for most cases, the MBS creates lung contours requiring little if any editing. The full process is semi-automatic and in the future, we aim to leverage the capabilities of artificial intelligence (AI) to auto segment the nodal CTV, CTV_TB, Heart and Contra_Breast as we found atlas-based segmentation less precise for some organs (*e.g.* the heart).

The initial step of manual placement of a medial tangent for the WB/PB/SIB plans adds time to the process; however, this is the current practice within the department and aligns with guidance provided for national breast trials. There are documented automatic potential solutions addressing this issue, some of these require radiopaque markers/wires on the skin at CT or outlining the CTV breast to guide field placement.^10,20,26^ Additionally, centres using these techniques usually have a manual contour review for acceptability prior to treatment planning. Manual tangent placement takes about 20 min and allows the person placing the beam to utilise all information at hand including the patient’s histology, operation notes, history and specific anatomy.

The final AP VMAT plans did not have flash incorporated into them to account for potential swelling/motion of the CTV breast/chest wall. This is in contrast to the clinical MP VMAT plans, which were robustly optimised considering flash of 1.5 cm motion of the CTV as previously described.^19^ The reason for this was mostly for time-saving testing efficiency and experience that we have that when adding flash, it does not significantly affect the dosimetry.^19^ Our process involves firstly producing a non-robust plan (quicker) and then the planner may wish to further manually tweak the plan optimisation before proceeding to copying this plan and optimising robustly. This is a time-saving method because robust optimisation over multiple data sets can be lengthy, so we try to find the right optimisation functions weights quickly before robustly optimising. This script version allows for the planner to tweak if required prior to proceeding to robust optimisation. If further testing shows similar good results with the VMAT AP non-robust plans, then another in-house “robust” script (already in use separately) can be joined to this script, so the plan can continue automatically to the robust optimisation stage (takes about 12 min to complete the robust optimisation step). Preliminary testing shows that in ~80% cases that this could be done.

In our study, dosimetric verification was not performed on the AP plans. However, currently we have introduced these scripted techniques into clinical practice and have not identified any change in dose verification pass/fail results. There is no reason why the dosimetry results should differ significantly as the scripted plans are merely automating a predominantly manual process.

The precise manual planning times were not specifically recorded but were based on estimations from allocated default planning times. As a result, the time is more than likely an overestimate of manual planning time. Despite this, it is clear from its use to the authors that this scripted solutionis more efficient for planners, especially those with less experience or work to a slower pace. The manual interaction time is less, allowing more focus on alternative tasks and planning errors can be reduced though automatic detection. Feedback from planners using the scripts have been very positive with overall recordings of auto plan times of ~30–45 min for WB/PB plans and ~2 h for SIB plans. Our aim is to release the next version of this script alongside a Raystation version upgrade to 12A and to reduce the allocated default planning times.

## Conclusion

Scripted semi-automatic planning of various modern breast techniques has been successfully introduced into a large busy NHS department using a commercial planning system. Scripted plans are similar to manual plans requiring little additional manual interaction for most cases and has the potential to reduce planning times significantly. The script in this paper is available upon reasonable request to the corresponding author. The code could potentially be altered accordingly to match the specific needs of a centres workflow.
